# Considerations on the Update of the Risk Assessment Document During the Pandemic State by COVID-19 in Italy

**DOI:** 10.3389/fpubh.2021.655927

**Published:** 2021-07-28

**Authors:** Luigi Cirrincione, Venerando Rapisarda, Caterina Ledda, Ermanno Vitale, Rosanna Provenzano, Emanuele Cannizzaro

**Affiliations:** ^1^Department of Health Promotion, Mother and Child Care, Internal Medicine and Medical Specialties “Giuseppe D'Alessandro”, University of Palermo, Palermo, Italy; ^2^Department of Clinical and Experimental Medicine, Occupational Medicine, University of Catania, Catania, Italy; ^3^Department of Juvenile and Community Justice, Rome, Italy

**Keywords:** COVID-19, risk assessment document, SARS-CoV-2, occupational medicine, prevention and protection measures

## Abstract

Immediately after the outbreak of the SARS-CoV-2 epidemic (which had risen to the level of a pandemic according to the World Health Organization), the question arose whether or not to update the risk assessment, which, as required by Legislative Decree 81/2008, with the consequent updating of the prevention measures. In light of these forecasts, we asked ourselves whether the risk of coronavirus infection should be taken into account by the employer by updating the risk assessment or not. An in-depth analysis of current legislation has led to the conclusion that the biological risk from SARS-CoV-2 is to be considered specific only in health-related activities, in other activities it can be considered exclusively generic or generic aggravated. The Risk Assessment Document can therefore only be integrated.

## Introduction

In December 2019, several cases of atypical pneumonia were detected in the city of Wuhan, Hubei Province in inland China. The first patients, it will be discovered later, had shown symptoms at the beginning of December, or even in mid-November. But it is on the last day of the year 2019, in a 41-year-old patient admitted 5 days earlier, that doctors officially identify a new virus called SARS-CoV-2 as the cause of this atypical pneumonia (1).

The initial outbreak took on considerable proportions first in China and then spread to the rest of the world; on 11 March 2020, the World Health Organization (WHO) declared that the new coronavirus SARS-CoV-2 infection can be considered a pandemic (2).

Current evidence suggests that SARS-CoV-2 spreads both directly, through close contact with infected people through secretions in the mouth and nose (saliva, respiratory secretions, or droplets), and indirectly through objects or surfaces contaminated with the same secretions (3). Following SARS-CoV-2 infection, the worst clinical effects, such as severe acute respiratory distress (ARDS), are more likely to occur in men than in women, possibly with comorbidities (4).

Italy was the first country within the Euro-Mediterranean area to experience the SARS-CoV-2 pandemic in a dramatic way. On 21 February the presence of the first “secondary” outbreak was detected, in which the transmission did not only concern people coming from risk areas. The first Italian patient identified, affected by COVID-19 was detected in Codogno (Lodi), not being linked to known outbreaks (1).

The Italian state has progressively implemented increasingly restrictive measures to avoid the spread of contagion, following the guidelines of the World Health Organization (5).

These restrictive measures include social distancing, community and communication strategies (6), the search for possible non-pharmaceutical interventions to mitigate contagion (7) until the national lockdown from 8 March 2020, i.e., the total blockage of all non-essential production and commercial activities until 06/2020 (8).

For production activities and essential services, which were unable to close, the “*Shared protocol for the regulation of measures to combat and contain the spread of the virus in the workplace*,” signed on 14 March 2020 (9), was published, which provided, among other things, for the use, where possible, of the working method defined as “agile or smart-working” to minimize contact between employees of the same company (10).

Subsequently, containment measures divided into organizational, environmental, and personal were applied when all production and commercial activities were reopened.

Organizational measures are general measures for the containment and management of the COVID-19 epidemiological emergency imposed by the competent authorities with the aim of minimizing the probability of being exposed to this new virus.

Among the organizational measures proposed to deal with the COVID-19 pandemic, it was considered of paramount importance:

- Minimize the entry of visitors into the workplace by limiting or restricting access to all company personnel, including employees.
- Preventing people with obvious flu-like symptoms from entering the company premises by having their body temperature taken, all staff, customers, suppliers, and external collaborators; not allowing those with a temperature above 37.5°C to enter the company premises.
- Inhibit access to traveling personnel from areas at increased risk for SARS-CoV-2, initially defined as “red zones,” or high class provinces based on the distribution of standardized cumulative incidence rates.
- Reducing the number of operators within individual confined spaces, making use of all possible home-based agile working modes and shifts in the workplace.
- Compose, where possible, two or more closed and independent working groups, to be alternated every 14 days to work alternately in the company and in smart-working (11).

Among the environmental measures aimed at reducing the risk of transmission of SARS-CoV-2 infection through contact with infected individuals, or through contact with inanimate or contaminated objects, equipment, and surfaces, the greatest emphasis was placed on cleaning and sanitizing work environments, These include the use of chemicals such as sodium hypochlorite-based disinfectants (0.1–0.5%), ethanol (62–71%), or hydrogen peroxide (0.5%), for an adequate contact time, providing more ventilation of the enclosed spaces following their use (12); that the use of physical means such as ultraviolet irradiation or the use of ozone for which enveloped viruses such as coronaviruses may be more sensitive (13, 14).

In November, following the increase in contagion due to what has been unanimously recognized as the “second pandemic wave,” new restrictive measures and selective limitations were issued through the Decree of the President of the Council of Ministers (now called D.P.C.M.) of 3 November 2020 in the Italian regions based on the different trend of the epidemic in the territory (15).

## Regulations

Legislative Decree 81/2008 in art. 268 of Title X (Exposure to biological agents) classifies biological agents in the following four groups (borrowed from European Directive 2000/54/EC):

- Group 1 biological agent: an agent that is unlikely to cause disease in human subjects;
- Group 2 biological agent: an agent that can cause disease in human subjects and pose a risk to workers; it is unlikely to spread to the community; effective prophylactic or therapeutic measures are usually available;
- Group 3 biological agent: an agent that can cause serious illness in human subjects and constitutes a serious risk to workers; the biological agent can spread in the community, but effective prophylactic or therapeutic measures are usually available;
- Group 4 biological agent: a biological agent that can cause serious diseases in human subjects and constitutes a serious risk for workers and may present a high risk of propagation in the community; effective prophylactic or therapeutic measures are not normally available (16).

In October 2019, Directive (EU) 2019/1833 amended Annex III to Directive 2000/54/EC by adding a number of biological agents to various groups, including Severe Acute Respiratory Syndrome Coronavirus (SARS-CoV) and Middle Eastern Respiratory Syndrome Coronavirus (MERS-CoV); in relation to the risk reduction measures to be put in place, it is clear that it was essential to include SARS-CoV-2 in one of these groups (17, 18).

**Figure 1:**
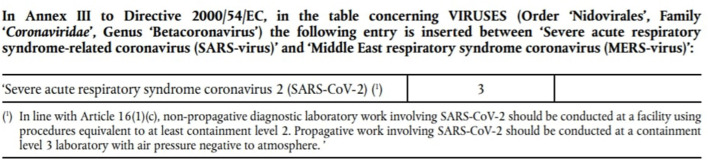
Annex to the new Commission Directive (EU) 2020/739 of 3 June 2020.

The new Commission Directive (EU) 2020/739 of 3 June 2020, in view of the most recent scientific evidence and clinical data available, as well as the opinions provided by experts representing all Member States, in order to continue to ensure adequate protection of workers' health and safety at work includes SARS-CoV-2 among the group 3 biological agents (Figure 1) (19, 20).

As was easy to predict, the emergency of the coronavirus has generated a series of delicate questions of interpretation which, in the field of labor law, also concern the application of the preventive discipline for the protection of health and safety at work provided for by Legislative Decree no. 81/2008.

On the contrary, on closer inspection, the management of the current emergency, precisely because it is intimately connected to the protection of people's health, risks creating real short circuits with the discipline of health and safety at work, subjected, as never before, to a tension that risks cracking some essential principles. More specifically, considerable uncertainties weigh heavily on the prevention measures to be adopted in production activities.

Immediately after the outbreak of the epidemic (which had risen to the level of a pandemic according to the World Health Organization), the problem arose as to whether or not the risk assessment should *be* updated, which, as provided for in Article 29, paragraph 3, of Legislative Decree no. 81/2008, “*must be immediately reworked, in accordance with the procedures”* referred to in Article 29, paragraph 3, of Legislative Decree no. 81/2008. 28, paragraphs 1 and 2, “*on the occasion of changes in the production process or work organization that are significant for the health and safety of workers, or in relation to the degree of technical evolution, prevention or protection or following significant accidents or when the results of health surveillance show the need to do so,”* (21) with the consequent updating of prevention measures. An assessment of the risks that Legislative Decree no. 81/2008 defines, consistently with the principle contained in the Framework Directive no. 89/391/EEC, as the “*global and documented assessment of all the risks for the health and safety of workers in the organization in which they work, aimed at identifying the appropriate prevention and protection measures and drawing up the programme of measures to guarantee the improvement of health and safety levels over time”* (art. 2, letter q; art. 28) (22).

In the light of these predictions, it was therefore questioned whether the risk of coronavirus infection should be taken into account by the employer by updating the risk assessment already carried out and the related document.

## Discussion

In order to answer this question, it is not enough to rely on the fact that the law requires “all” risks to be assessed, since the legislator has clearly indicated that these must be “all” risks present within the organization in which the workers operate, i.e., the specific risks that are connected to the structural, instrumental, procedural, and rules that the employer has conceived and implemented for the pursuit of its production purposes.

It is clear that, manifesting itself through contact between people, the biological risk arising from SARS-CoV-2 can well creep into production organizations where people are working.

With the exception of some specific work activities, such as those carried out in health and hospital services, in other cases, far from becoming a specific occupational risk, the biological risk deriving from SARS-Cov2 is identifiable as a generic risk, which does not derive from the specific organization work, but rather makes use of the complex system of personal relationships on which it is based to manifest and spread, often coming from outside the working environment itself: this is the case of a worker who becomes infected in an environment outside the company and, going to work on it, introduces the virus.

A separate reflection should be made for those occupational contexts that subject workers to particularly intense psychophysical stress, involving for example shift work, or in cases where social distancing is not feasible, such as in professional team sports, such as football or water polo, in which we have seen an increase in the incidence of infection (23–27).

Without prejudice to the nature of the specific biological risk of some sectors such as those mentioned above, it cannot be denied that particular business contexts can lead to an increase in the level of exposure to the risk of contagion, compared to the socially accepted level in the community to which the worker belongs, configuring what gives some are defined as an aggravated generic risk (28). In this case, in fact, the worker is more exposed, both in terms of intensity and frequency, to the pandemic risk, involving to a greater extent those who work during the emergency period. And although the notion of aggravated risk is not expressly contained in the law, it is unanimously recognized by jurisprudence as a reference to a risk that has an etiological link with work.

Taking into account what has been said up to now, the employer, although not having to proceed with the development of a new risk assessment, will have to proceed with the adaptation of those measures already identified (regarding the possible presence and influx of hygiene, adoption and supply of the identified PPE) that reflect the protection indications already described in general terms by emergency regulations, safety protocols and national D.P.C.M., to be adapted only to the specific activity.

To be understood as the employer, while not having to bear the application of the aforementioned Title X of the Legislative Decree 81/2008 on exposure to biological agents (since, since the coronavirus is not an ontologically inherent biological agent in that organization, it gives rise to a biological and non-specific risk), it will have to guarantee its assignment in the company of the prevention measures dictated by the public authority, however it is up to him to evaluate and decide how to adopt them in his own company where they present margins of discretion.

## Conclusions

The above considerations therefore lead to the conclusion that a new risk assessment should not be carried out, but it is appropriate to formalize the action through acts that take into account the attention paid to the problem in terms of measures, in any case adopted and adoptable from point from a technical, organizational, and procedural point of view, as well as the PPE deemed necessary, in implementation of the national, regional, and local indications of the bodies in charge which, as already mentioned, go hand in hand with the ordinary company prevention measures, without being able to modify them and maintaining a distinct nature and specific purpose.

## Data Availability Statement

The original contributions presented in the study are included in the article/supplementary material, further inquiries can be directed to the corresponding author.

## Author Contributions

EC conceived the article and contributed in its drafting. LC carried out the bibliographic research and contributed in its drafting. VR, EV, and RP carried out the bibliographic research. CL participated in bibliographic research and reviews process of the article. All authors contributed to the article and approved the submitted version.

## Conflict of Interest

The authors declare that the research was conducted in the absence of any commercial or financial relationships that could be construed as a potential conflict of interest.

## Publisher's Note

All claims expressed in this article are solely those of the authors and do not necessarily represent those of their affiliated organizations, or those of the publisher, the editors and the reviewers. Any product that may be evaluated in this article, or claim that may be made by its manufacturer, is not guaranteed or endorsed by the publisher.
